# Forecasts Aid HABs Response

**DOI:** 10.1289/ehp.119-a510

**Published:** 2011-12-01

**Authors:** Kris S. Freeman

**Affiliations:** Kris S. Freeman has written for *Encarta* encyclopedia, NIH, ABCNews.com, and the National Park Service. Her research on the credibility of online health information appeared in the June 2009 *IEEE Transactions on Professional Communication*.

Large accumulations of algae, or harmful algal blooms (HABs), can release toxins and contribute to oxygen-depleted “dead zones” in waters, causing human health problems and disrupting food webs.^1^ Costs associated with closed fisheries and beaches caused by marine HABs total at least $82 million annually^2^; freshwater HABs can complicate water treatment and decrease recreational revenue, accounting for millions more in costs.^3^ Now consortia of researchers across the country are developing forecasts to help local public health officials better monitor and respond to HABs.

Anthropogenic nutrient enrichment and a warming climate are contributing to increased frequencies, intensities, and distributions of freshwater cyanobacteria HABs worldwide.^1^^,^^4^^,^^5^ When cyanobacteria covered much of Lake Erie’s western basin in July 2011, levels of the cyanotoxin microcystin-LR reached 1,000 µg/L in near-record^6^ water temperatures averaging just above 77°F.^7^ An Ohio Department of Health representative reports nine probable cases of algal-caused illnesses in Ohio in 2011 (unpublished data); however, many cases of illness attributable to HABs, especially milder cases, are probably never reported.

Although reports of marine HABs—which include blooms of the dinoflagellates *Karenia brevis* and *Alexandrium* spp. and the diatoms *Pseudo-nitzschia* spp.—appear to be increasing,^1^ researchers lack the long-term data sets needed to prove incidences really are on the rise,^8^ according to Barbara Kirkpatrick, a senior scientist at Mote Marine Laboratory in Sarasota, Florida. “The more we look for HABs, the more we find them,” she says. “The more we populate along our coastlines, the greater the impact of HABs on people.”

The health effects resulting from exposure to HAB toxins can include gastric, respiratory, and neurologic impacts; acute exposure to some HAB toxins can cause death. Exposure can occur through consumption of fish, shellfish, or crustaceans that accumulate the toxins when feeding on algae. Commercial shellfish operations are now routinely monitored for HABs, but controlling recreational harvests is more difficult.^1^ “We only have toxic shellfish poisonings in Florida when a visitor wanders on the beach and collects clams or oysters from an area not open for harvesting,” says Andrew Reich, coordinator of the Florida Department of Health Aquatic Toxins Program.

**Figure:**
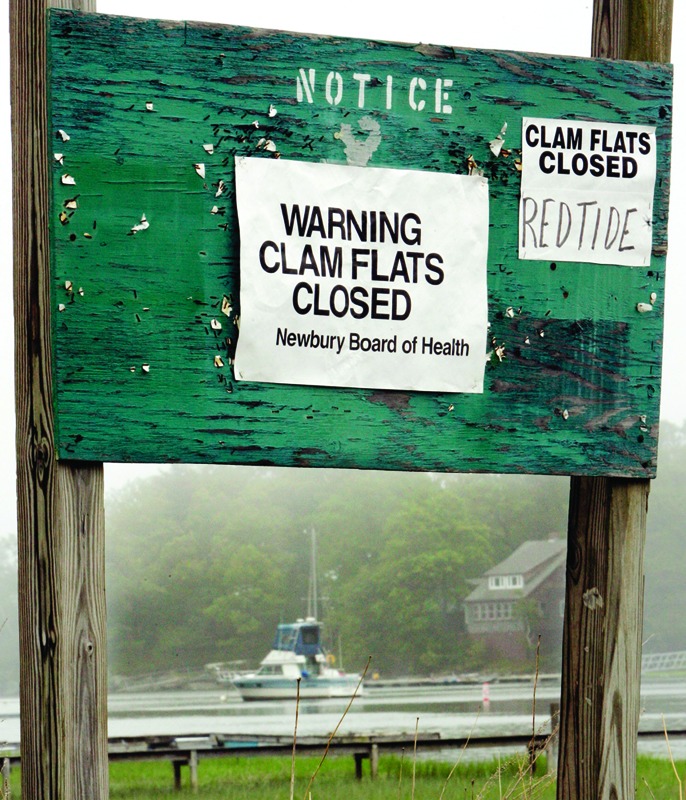
A Massachusetts sign warns of the historic A. fundyense outbreak that hit the New England coast in 2005. ©Brian Snyder/Reuters/Corbis

A multiagency con-sor-tium of re-searchers led by the National Ocean-ic and Atmospheric Admin-istration (NOAA) is working to improve prediction and monitoring of HABs.^9^ In 2004 NOAA initiated a forecasting system for *K. brevis* in the Gulf of Mexico that uses satellite imagery, field observations, and buoy data to provide information on HAB locations, extent, and potential for change in size or location. Woods Hole Ocean-ographic Institution (WHOI), supported by NOAA, led an effort to use computer models to successfully predict HABs of *Alexandrium fundyense* in the Gulf of Maine in 2008.^10^ NOAA is now operating a demonstration forecast project for cyanobacteria HABs in Lake Erie and is coordinating development of forecasts for HABs of the dinoflagellate *Karlodinium veneficum* in the Chesapeake Bay and of *Pseudo-nitzschia* on the Washington coast.

Detecting HABs can be tricky. Not all blooms produce toxins, even though they may have unpleasant tastes and odors. “Visible blooms may be harmless, and harmful blooms may be almost invisible,” says Sherwood Hall, a marine toxin specialist at the Center for Food Safety and Applied Nutrition of the U.S. Food and Drug Administration. To further complicate management, conditions that cause algal blooms to produce toxins can vary dramatically among different organisms.

Forecasting doesn’t eliminate the need for shellfish resource managers to test and monitor toxicity, but it can help them do so more effectively, says WHOI senior scientist Dennis McGillicuddy, a member of the team that developed HAB forecasting methods for the Gulf of Maine. It can also assist local public health departments in their responses to HABs. The NOAA forecasts are “wonderful for us in public health,” says Reich, who advises Florida’s 67 local health departments on HAB response. “Generally, if they find a HAB offshore, and there’s a circulation pattern or wind pattern blowing it onshore, we have two or three days’ warning, so we have time to gear up.”

## References

[1] Ramsdell JS, et al., eds. (2005). HARRNESS. Harmful Algal Research and Response: A National Environmental Science Strategy, 2005–2015. http://tinyurl.com/3pk8s8j.

[2] NCCOS. Economic Impacts of Harmful Algal Blooms (HABs) [website]. Silver Spring, MD:National Centers for Coastal Ocean Science, National Oceanic and Atmospheric Administration (16 Feb 2011). Available: http://tinyurl.com/2dv779r [accessed 20 Oct 2011]

[3] Dodds WK (2009). Eutrophication of U.S. freshwaters: analysis of potential economic damages.. Environ Sci Technol.

[4] Paerl HW (2011). Controlling harmful cyanobacterial blooms in a world experiencing anthropogenic and climatic-induced change.. Sci Total Environ.

[5] Paerl HW, Paul VH. Climate change: links to global expansion of harmful cyanobacteria. Water Res; http://dx.doi.org/10.1016/j.watres.2011.08.002 [online 18 Aug 2011]. 10.1016/j.watres.2011.08.002 21893330

[6] NWS. Lake Erie Water Temperatures [website]. Buffalo, NY:Weather Forecast Office Buffalo, National Weather Service (7 Jan 2011). Available: http://tinyurl.com/cfflmud [accessed 20 Oct 2011]

[7] Center of Excellence for Great Lakes and Human Health. Experimental Lake Erie Harmful Algal Bloom Bulletin. Ann Arbor, MI:Great Lakes Environmental Research Laboratory, National Ocean Service (28 Jul 2011). Available: http://tinyurl.com/3e2jof2 [accessed 20 Oct 2011]

[8] Efforts to improve HAB data sets include a five-year effort by the Centers for Disease Control and Prevention to collect and store HAB data from 13 states. Harmful Algal Bloom-Related Illness Surveillance System (HABISS). Atlanta, GA:Harmful Algal Blooms Program, U.S. Centers for Disease Control and Prevention. Available: http://tinyurl.com/4yf7oq2 [accessed 20 Oct 2011]

[9] NOAA. Developmental Harmful Algal Bloom Forecast Projects [website]. Washington, DC:National Oceanic and Atmospheric Administration (1 Jun 2011). Available: http://tinyurl.com/5sgxuhf [accessed 20 Oct 2011]

[10] WHOI. Researchers Successfully Forecast 2008 Red Tide [press release]. Woods Hole, MA:Woods Hole Oceanographic Institution (8 Aug 2008). Available: http://tinyurl.com/7stvpua [accessed 20 Oct 2011]

